# Clinical impact of postoperative interval until adjuvant chemotherapy following curative gastrectomy for advanced gastric cancer

**DOI:** 10.7150/jca.58154

**Published:** 2021-08-13

**Authors:** Yusuke Takashima, Shuhei Komatsu, Keiji Nishibeppu, Jun Kiuchi, Takuma Ohashi, Hiroki Shimizu, Tomohiro Arita, Yusuke Yamamoto, Hirotaka Konishi, Ryo Morimura, Atsushi Shiozaki, Yoshiaki Kuriu, Hisashi Ikoma, Takeshi Kubota, Hitoshi Fujiwara, Kazuma Okamoto, Eigo Otsuji

**Affiliations:** Division of Digestive Surgery, Department of Surgery, Kyoto Prefectural University of Medicine, 465 Kajii-cho, Kawaramachihirokoji, Kamigyo-ku, Kyoto, Japan.

**Keywords:** adjuvant chemotherapy, S-1, early initiation, cumulative dose, gastric cancer

## Abstract

**Background:** Adjuvant chemotherapy (AC) following curative gastrectomy for stage II/III gastric cancer (GC) is recommended in Japan. However, for various reasons, patients cannot always start AC at the appropriate time. This study was designed to investigate the effect of the postoperative interval until adjuvant chemotherapy (PIAC) and cumulative S-1 dose on prognosis.

**Methods:** Between 2008 and 2014, consecutive 81 GC patients who underwent postoperative S-1 monotherapy were enrolled in this study.

**Results:** Postoperative complications of Clavien-Dindo grade II or higher and postoperative peak C-reactive protein of 8.1 mg/dl or higher were significantly associated with delayed AC. The cut-off value of PIAC selected to most effectively stratify prognosis was 7 weeks. For relapse-free survival (RFS), patients with PIAC ≥ 7 weeks had an insignificantly poorer prognosis than those with PIAC < 7 weeks. A multivariate analysis showed that PIAC ≥ 7 weeks [*p* = 0.024; hazard ratio (HR) 2.45] and the cumulative S-1 dose/body surface area (BSA) ≥ 12,000 mg/m^2^ [*p* = 0.004; HR 3.27] were independent prognostic factors. In patients with the cumulative S-1 dose/BSA ≥ 12,000 mg/m^2^, there were no prognostic differences between patients with and without PIAC ≥ 7 weeks.

**Conclusions:** Seven weeks after surgery could be a limit indicator starting AC. A cumulative S-1 dose/BSA of more than 12,000 mg/m^2^ might be a key dose for diminishing the poor prognostic effects of delaying AC.

## Introduction

Gastric cancer (GC) is the fourth most commonly diagnosed cancer, and the third most common cause of cancer-related death worldwide 1. Treatments for GC has been enhanced by improvements in surgical procedures and perioperative management 2-6. Although surgical resection mainly involves macroscopic tumor resection and lymphadenectomy, the oncologic effect of surgical treatment is often limited to local control. Therefore, perioperative therapy has been recommended to clear remaining microscopic metastasis 7.

The ACTS-GC (Adjuvant Chemotherapy Trial of TS-1 for Gastric Cancer) study and its 5-year follow-up results demonstrated that adjuvant chemotherapy (AC) with S-1 monotherapy following curative gastrectomy with lymphadenectomy significantly improved relapse-free survival (RFS) and overall survival rates (OS). Therefore, AC following curative gastrectomy has been recommended as the standard treatment in Japan for stage II/III gastric cancer since 2014 8, 9.

The ACTS-GC study proved the beneficial prognostic effect of starting S-1 treatment within 6 weeks of undergoing curative surgery. However, patients cannot always start AC at the appropriate time following surgery due to various clinical issues that can result in delayed recovery. The prognostic effect of any delay with regard to AC remains controversial 10, 11. Even if the prognosis may be affected by a delay in starting AC, until now there has been no definite cut-off value for the postoperative duration following curative surgery with which to stratify the prognosis 12. Moreover, it is not clear whether a sufficient cumulative S-1 dose during AC could affect the prognosis or diminish the poor prognostic effect resulting from a delay in starting AC.

In this study, we investigated the clinical significance of the postoperative interval until adjuvant chemotherapy (PIAC) and the cumulative S-1 dose, with particular reference to the prognosis. The results of our study suggest a crucial indicator starting AC. Our study also suggested that the cumulative S-1 dose might be key to diminishing any poor prognostic effect, even if there is a delay in starting AC.

## Methods

### Patients and surgical procedures

This study was approved by the Kyoto Prefectural University of Medicine, Japan, and was therefore performed in accordance with the ethical standards laid down in the Declaration of Helsinki. Written informed consent was obtained from all patients to participate in the research. A total of 214 consecutive patients underwent curative gastrectomy with lymphadenectomy for stage II or III GC at our institute between January 2008 and December 2014. Of these 214 patients, we excluded patients who refused AC because of old age, comorbidities and other private reasons and patients who received other AC regimens, and patients who received AC at the other hospitals after surgery. As a result, 95 patients received S-1 monotherapy as AC. Moreover, we excluded 14 patients from the study because patients had neoadjuvant chemotherapy (n = 2), insufficient follow-up (n = 1), remnant GC (n = 3), and multiple carcinomas (n = 8). As a result, we retrospectively investigated 81 consecutive patients who received S-1 AC.

Patients with clinical stage IA (clinical T1 and clinical N0 GC) underwent D1+ lymphadenectomy, while those with clinical stage IIA or higher underwent D2 or D2+ lymphadenectomy. All patients underwent macroscopic curative resection (R0); resected specimens were examined by pathologists and evaluated in accordance with the Japanese classification of GC 13. Dissected lymph nodes were fixed in buffered formalin and embedded in paraffin prior to pathological examination. Pathologists in our institution examined embedded lymph nodes by sectioning slices in the plane of the largest node dimension to confirm the presence of metastasis. Clinicopathological findings from these patients were determined retrospectively on the basis of their hospital records.

### Follow-up after curative gastrectomy followed by AC

Postoperative follow-up was performed in the outpatient clinic every three months following surgery. Blood chemistry was also measured every three months. Endoscopic examinations were performed annually, and computed tomography (CT) examinations were performed every three-to-six months for five years after surgery. Median follow up period was 5.1 years (interquartile range, 3.4 to 7.0 years) and the complete follow-up rate for 5 years was 93.8% (76/81 patients).

In this study, T1N2-3 and T3N0 GC patients were enrolled although these sub-stage patients were not included in ACTS-GC trial. The reasons are that patients with T1N2-3 had poor prognosis and T3N0 was not included in stage II when ACTS-GC trial was planned. Although there is no RCT based evidence demonstrating a survival benefit, we previously demonstrated that T3 GC patients more strongly correlated with the presence of lymphatic spread and peritoneal recurrence than T2 GC patients 14 and the prognosis for a specific subgroup of T3N0 GC patients was significant worse 15. In addition, the other current studies demonstrated that AC might improve the prognosis for T3N0 and T1N2-3 GC patients 16, 17. Therefore, we performed AC for these patients after giving sufficient information.

AC was begun in the outpatient clinic following discharge. The dose of S-1 was determined according to a patient's body surface area (BSA). Specifically, patients with BSA < 1.25 m^2^ received 80 mg per day; patients with 1.25 m^2^ ≤ BSA < 1.5 m^2^ received 100 mg per day; and patients with 1.5 m^2^ ≤ BSA received 120 mg per day. In order to modify the difference of BSA, the adjusted cumulative S-1 dose was calculated by dividing cumulative S-1 dose (mg) by BSA (m^2^). The cumulative S-1 dose received by each patient during AC was calculated from their hospital records.

### Definition of cut-off values

The Mann-Whitney U test was used to determine the optimal cut-off values as mentioned previously. Regarding the cut-off value of age and body mass index (BMI), the cut of value of 65 years old has been often used previously and BMI < 18.5 has been used as an underweight 18-20. Concerning Prognostic nutrition index (PNI), patients who had a PNI score < 40 were considered to have severe malnutrition according to a previous study 21, 22. Regarding the cut-off value of postoperative peak C-reactive protein (CRP), we used 8.1 as an optimal cut-off value by ROC analysis in our cohort for delayed initiation of AC (PIAC ≥ 7 weeks) (AUC = 0.60).

### Statistical analysis

Statistical analyses were conducted using JMP version 10 (SAS Institute Inc., Cary, NC). The Mann-Whitney U test for unpaired data comprising continuous variables was used to compare clinicopathological variables. For the analysis of survival, Kaplan-Meier survival curves were constructed for groups based on univariate predictors, and differences between the groups were tested using a generalized Wilcoxon test. The Cox proportional hazards model was used for further evaluations of multivariate survival analysis. A *p*-value < 0.05 was considered statistically significant.

## Results

### Clinicopathological characteristics of GC patients who received curative gastrectomy followed by S-1 AC

**Table 1** shows the clinical characteristics of GC patients who received curative gastrectomy followed by S-1 AC. The median age of the patients was 63.1 years. Of 81 patients, 51 (63%) were male and 30 (37%) were female; 30 patients were at pStage IIA, 17 patients were at pStage IIB, 17 patients were at pStage IIIA, and 17 patients were at pStage IIIB/IIIC. Total gastrectomy was performed in 34 patients (42%), distal gastrectomy in 45 patients (56%), and proximal gastrectomy was performed in 2 patients (2%) for curative resection dependent on the location of the tumor. A total of 20 patients (25%) underwent D1+ lymphadenectomy based on their clinical stage, while the remaining patients performed D2 or D2+ lymphadenectomy 13.

### Cut-off value of PIAC to stratify the prognosis and correlation between PIAC and clinicopathological factors

**Figure 1:**
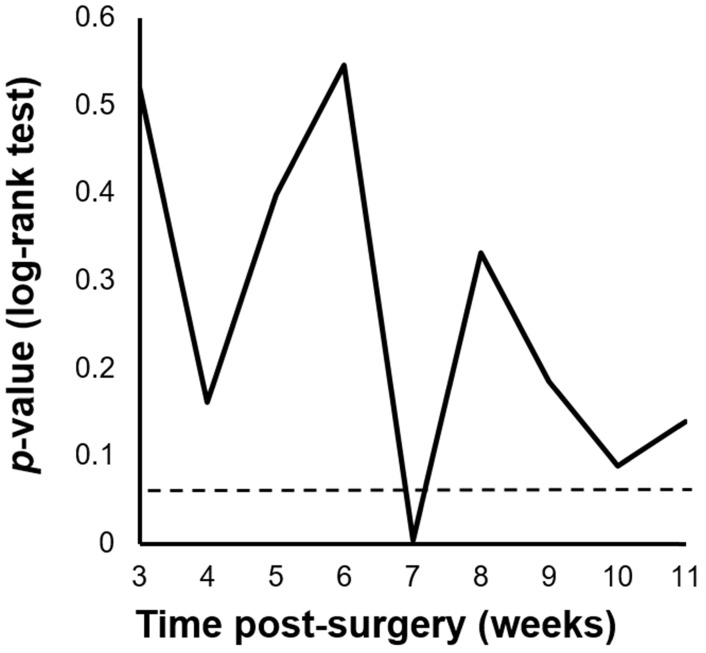
** Cut-off values of the postoperative duration until adjuvant chemotherapy (PIAC).** The cut-off value of PIAC to stratify relapse-free survival rates the most effectively in pStage II/III gastric cancer was 7 weeks post-surgery (*p* = 0.004).

**Figure 2:**
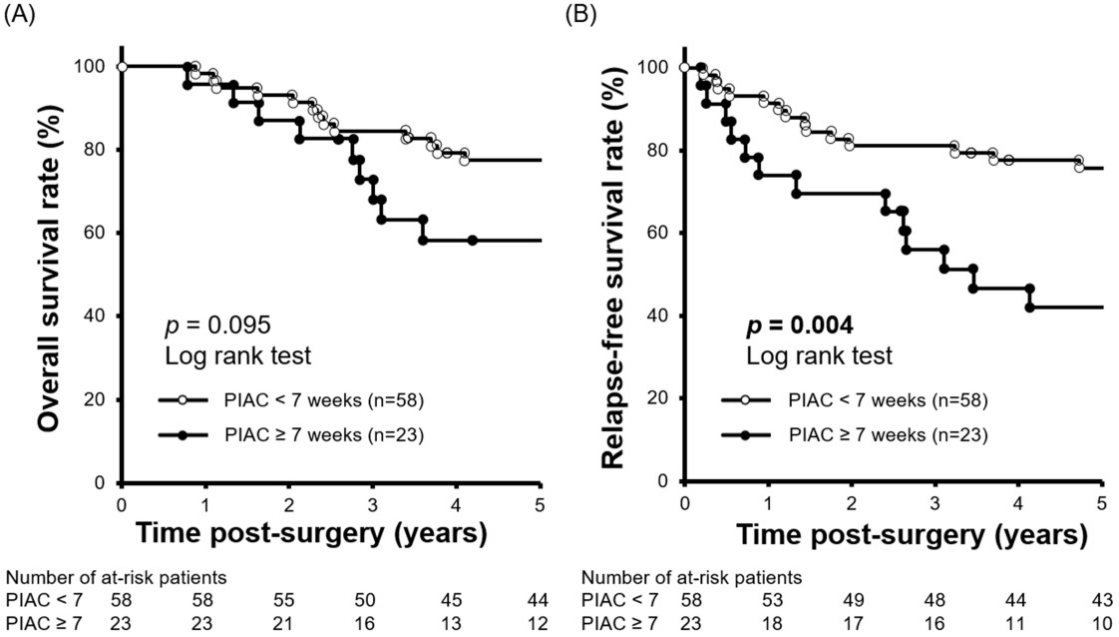
** Comparison of 5-year relapse-free survival rates between patients with PIAC ≥ 7 weeks and patients with PIAC < 7 weeks. (A)** Patients with PIAC ≥ 7 weeks had a poorer overall survival rate than patients with PIAC < 7 weeks (*p* = 0.095; 5-year OS: 58.3% vs. 77.4%). **(B)** Patients with PIAC ≥ 7 weeks had a significantly poorer relapse-free survival rate than patients with PIAC < 7 weeks (*p* = 0.004; 5-year RFS: 42.4% vs. 75.7%).

We performed a minimum *p*-value analysis for 5-year RFS using various cut-off values for PIAC, as shown in **Figure 1**. The cut-off value of 7 weeks post-surgery was confirmed to be the upper-limit to stratify the prognosis (*p* = 0.004; 5-year RFS: PIAC ≥ 7 weeks vs. PIAC < 7 weeks; 42.4% vs. 75.7%). Although there were no significant differences in 5-year OS, patients in the PIAC ≥ 7 weeks group had a poorer prognosis than those in the PIAC < 7 weeks group (*p* = 0.095; 5-year OS: PIAC ≥ 7 weeks vs. PIAC < 7 weeks; 58.3% vs. 77.4%) (**Figure 2**).

### Cut-off value of cumulative S-1 dose to stratify the prognosis and combined survival curves using PIAC and adjusted cumulative S-1 dose factors

To clarify the clinical effect of an adjusted cumulative S-1 dose, we performed a minimum p-value analysis for 5-year RFS using various cut-off values of the adjusted cumulative S-1 dose (data not shown). A cut-off value of 12,000 mg/m^2^ was confirmed to stratify the prognosis most (*p* = 0.002; 5-year RFS: adjusted cumulative S-1 dose ≥ 12,000 mg/m^2^ vs. < 12,000 mg/m^2^; 82.7% vs. 49.5%).

**Figure 3:**
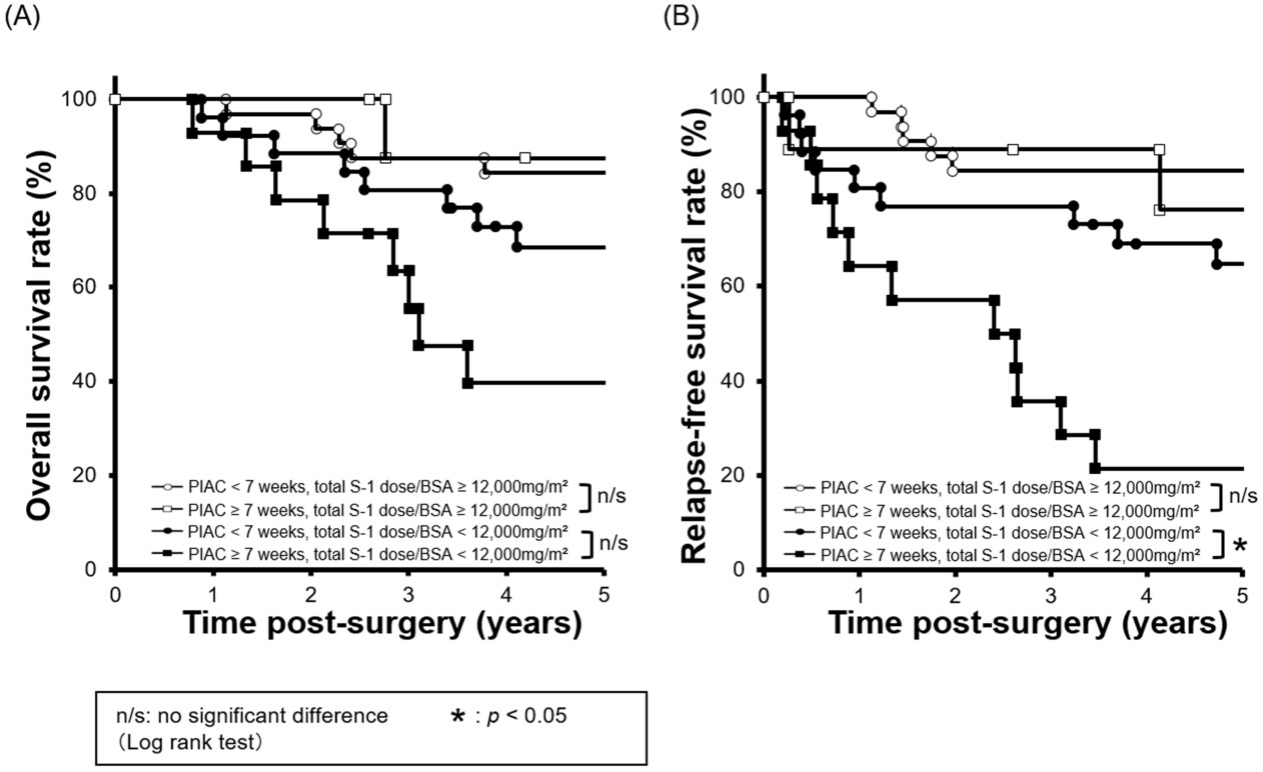
** Combined survival curves using PIAC and cumulative S-1 dose factors.** The cohort was divided into four groups: PIAC ≥ 7 weeks and total S-1 dose/BSA ≥ 12,000 mg/m^2^; PIAC < 7 weeks and total S-1 dose/BSA ≥ 12,000 mg/m^2^; PIAC ≥ 7 weeks and total S-1 dose/BSA < 12,000 mg/m^2^; and PIAC < 7 weeks and total S-1 dose/BSA < 12,000 mg/m^2^. **(A)** There were no significant prognostic differences between the PIAC ≥ 7-weeks group and the PIAC < 7-weeks group for 5-year OS in patients with total S-1 dose/BSA < 12,000 mg/m^2^ (*p* = 0.070; 5-year OS: PIAC ≥ 7 weeks vs. PIAC < 7 weeks; 39.7% vs. 68.6%) and in patients with total S-1 dose/BSA ≥ 12,000 mg/m^2^ (*p* = 0.743; 5-year RFS: PIAC ≥ 7 weeks vs. PIAC < 7 weeks; 87.5% vs. 84.4%). **(B)** For patients with total S-1 dose/BSA < 12,000 mg/m^2^, there were significant prognostic differences between the PIAC ≥ 7-weeks group and the PIAC < 7-weeks group for RFS (*p* = 0.007; 5-year RFS: PIAC ≥ 7 weeks vs. PIAC < 7 weeks; 21.4% vs. 64.7%). However, for patients with total S-1 dose/BSA ≥ 12,000 mg/m^2^, there were no prognostic differences between the PIAC ≥ 7-weeks group and the PIAC < 7 weeks group for RFS (*p* = 0.630; 5-year RFS: PIAC ≥ 7 weeks vs. PIAC < 7 weeks; 76.2% vs. 84.4%).

Next, we compared survival curves between four groups: 1) PIAC ≥ 7 weeks / adjusted cumulative S-l dose ≥ 12,000 mg/m^2^; 2) PIAC < 7 weeks/adjusted cumulative S-l dose ≥ 12,000 mg/m^2^; 3) PIAC ≥ 7 weeks/adjusted cumulative S-l dose < 12,000 mg/m^2^; and 4) PIAC < 7 weeks/adjusted cumulative S-l dose < 12,000 mg/m^2^. With regard to patients who received the adjusted cumulative S-l dose < 12,000 mg/m^2^, there was a significant prognostic difference between the PIAC ≥ 7 weeks group and the PIAC < 7 weeks group for RFS (*p* = 0.007; 5-year RFS: PIAC ≥ 7 weeks vs. PIAC < 7 weeks; 21.4% vs. 64.7%). However, in patients who received the adjusted cumulative S-1 dose > 12,000 mg/m^2^, there was no prognostic differences between the PIAC ≥ 7 weeks group and PIAC < 7 weeks group for RFS (*p* = 0.630; 5-year RFS: PIAC ≥ 7 weeks vs. PIAC < 7 weeks; 76.2% vs. 84.4%) (**Figure 3**). In addition, we compared 5-year RFS by using various cut-off number of the adjusted cumulative S-1 dose for patients in PIAC ≥ 7 weeks group. When the cut-off number of the adjusted cumulative S-1 dose was more than 12,000 mg/m^2^, there was no prognostic differences between patients who had received the cut-off number or less S-1 dose and that who had done more (**Table 2**).

### Comparison of PIAC with clinicopathological factors

Next, we evaluated correlations between PIAC and clinicopathological factors using the Mann-Whitney U test. As shown in **Table 3**, a high peak in the post-operative CRP cut-off value of 8.1 mg/dl or above was significantly associated with a delay in starting S-1 AC (*p* = 0.007). In addition, the incidence of postoperative complications (Clavien-Dindo classification ≥ II) was significantly linked to longer PIAC (*p* = 0.026). There were no other significant differences between the groups with regard to other clinicopathological factors.

### Univariate and multivariate analysis using Cox's proportional hazard model

To elucidate the prognostic factors for 5-year RFS, univariate and multivariate analysis using Cox's proportional hazard model were performed. As shown in **Table 4**, age, sex, BMI, histological type, a postoperative peak CRP of ≥ 8.1 mg/dl, complications of Clavien-Dindo grade II or higher, pathological stage, an adjusted cumulative S-1 dose, and PIAC were selected as clinical variables. The multivariate analysis showed that PIAC ≥ 7 weeks [p = 0.024; hazard ratio (HR) 2.45 (95% CI: 1.13-5.29)], adjusted cumulative S-1 dose/BSA < 12,000 mg/m^2^ [p = 0.004; HR 3.27 (95% CI: 1.13-5.29)] and pathological advanced stage [p = 0.005; HR 3.08 (95% CI: 1.41-7.22)] were independent prognostic factors.

## Discussion

There have been few reports of prognostic effects with regard to PIAC and the extent of S-1 treatment in AC 11, 12, 23. In this study, we clearly demonstrated that a PIAC ≥ 7 weeks and a cumulative S-1 dose/BSA of more than 12,000 mg/m^2^ were independent prognostic factors in GC patients undergoing AC following curative gastrectomy. Moreover, in patients who received a cumulative S-1 dose/BSA of more than 12,000 mg/m^2^, we showed that there were no prognostic differences between patients who had a PIAC of more than or less than 7 weeks. PIAC ≥ 7 weeks. Our results strongly suggested that a cumulative S-1 dose/BSA of more than 12,000 mg/m^2^ is a crucial indicator and might be the key dose for diminishing the poor prognostic effects arising from a delay in AC.

Concerning PIAC, we clearly demonstrated that 7 weeks was the best cut-off value to stratify the prognosis of patients with pStage II/III gastric cancer (*p* = 0.004; 5-year RFS: PIAC ≥ 7 weeks vs. PIAC < 7 weeks; 42.4% vs. 75.7%). From an oncological perspective, to eliminate microscopic metastasis it appears that AC should be started immediately following curative gastrectomy. PIAC has been reported to be an independent prognostic factor in other types of cancer, with various cut-off values suggested, including 12 weeks 10 and 8 weeks in colon cancer 24, 25 and 13 weeks (91 days) in breast cancer 26. With regard to gastric cancer, various cut-off values of PIAC have been reported as prognostic factors, ranging from 4 to 8 weeks 12, 27-30. Our PIAC cut-off value of 7 weeks could also be a candidate indicator. Future studies are warranted, and big data analysis might be needed to determine the best cut-off value for clinical settings.

Regarding the cumulative S-1 dose/BSA, we also demonstrated that a cumulative S-1 dose/BSA of less than 12,000 mg/m^2^ was an independent prognostic factor for 5-year RFS [*p* = 0.024; HR 2.45 (95% CI: 1.13-5.29)]. The Japanese phase 3 randomized trial (JCOG1104 [OPAS-1]) revealed that four courses of S-1 AC were inferior to eight courses of S-1 AC for achieving RFS of pStage II gastric cancer, highlighting the importance of the duration of AC 31. Fujitani et al. also reported that S-1 AC with a duration of more than 6 months could have a prognostic impact in patients with GC 11. There results could be a crucial indicator of the importance of S-1 AC duration. However, the S-1 dose intensity in each patient is considerably different in clinical practice, and is indeed not as high as shown in previous reports 8, 32, 33. Therefore, we suggest that the cumulative S-1 dose is also a pivotal factor, in addition to the PIAC of S-1.

The most striking finding in the present study was that there were no prognostic differences between the PIAC ≥ 7-weeks group and the PIAC < 7-weeks group for RFS in patients with a cumulative S-1 dose/BSA ≥ 12,000 mg/m^2^ (*p* = 0.630; 5-year RFS: PIAC ≥ 7 weeks vs. PIAC < 7 weeks; 76.2% vs. 84.4%). Specifically, these results suggested that a cumulative S-1 dose/BSA of more than 12,000 mg/m^2^ might diminish the poor prognostic effect associated with a delay in AC. Similar results were also reported that S-1 AC of more than 6 months 11 and S-1 AC with a relative dose intensity of more than 64.6% 12 have more prognostic impact than PIAC. Although which factors have the optimal impact on prognosis is unclear, we enhance on the cumulative S-1 dose. As shown by our results, patients could not always begin AC at the appropriate time because of various clinical issues, such as a high serum CRP level or postoperative complications. To avoid postoperative complications 34 and achieve low postoperative CRP levels 35, less invasive surgery is a pivotal surgical goal from a prognostic perspective, Nevertheless, if these issues are encountered and AC cannot be initiated with sufficient S-1 dose intensity, administering high cumulative S-1 dose treatment even after a long PIAC could be a valuable strategy that could potentially rescue high-risk patients with these issues.

This study had some limitations. First, the results were obtained from a retrospective evaluation of a small number of patients at a single institute. Second, we excluded patients who refused AC because of old age, comorbidities and other private reasons and patients who received other AC regimens, and patients who received the AC at the other hospitals after surgery, which would be possible bias. A large-scale prospective study and multi-center cohort study is necessary to confirm the significance of PIAC and cumulative S-1 dose.

## Figures and Tables

**Table 1:** Clinicopathological characteristics of patients who received S-1 monotherapy as adjuvant chemotherapy

Variable	n	%
Total	81	
**Sex**		
Male	51	63%
Female	30	37%
**Age (years)**		
≥ 65	43	53%
< 65	38	47%
**BMI ^a^ (kg/m^2^)**		
≥ 18.5	73	90%
< 18.5	8	10%
**Tumor location**		
Upper	26	32%
Middle	35	43%
Lower	20	25%
**pT category^b^**		
T1	6	7%
T2	12	15%
T3	39	48%
T4	24	30%
**pN category^b^**		
N0	27	33%
N1	12	15%
N2	24	30%
N3	18	22%
**pStage^b^**		
IIA	30	37%
IIB	17	21%
IIIA	17	21%
IIIB/IIIC	17	21%
**Histological type**		
Differentiated	32	40%
Undifferentiated	49	60%
**Operation**		
Total gastrectomy	34	42%
Distal gastrectomy	45	56%
Proximal gastrectomy	2	2%
**Lymphadenectomy**		
D1+	20	25%
D2 or D2+	61	75%

^a^ BMI: body mass index; ^b^ Japanese Classification of Gastric Carcinoma.

**Table 2:** Prognostic effect in patients in the PIAC ≥ 7 weeks group depending on different cut-off of an adjusted cumulative S-1 dose

Cumulative S-1 dose/BSA (mg/m^2^)	*p*-value (Log rank test)
9,000	**0.010**
10,000	**0.010**
11,000	**0.039**
12,000	**0.012**
13,000	0.107
14,000	0.080
15,000	0.080
16,000	0.180
17,000	0.180
18,000	0.165
19,000	0.165
20,000	0.165

**Table 3:** Comparison of PIAC with clinicopathological factors

Variable	n	PIAC ^a^ (days)	*p*-value ^b^
**Sex**		(mean ± SD*^c^*)	
Male	51	(42.6 ± 13.6)	0.747
Female	30	(45.8 ± 24.2)	
**Age (years)**			
≥ 65	43	(40.7 ± 12.9)	0.895
< 65	38	(41.4 ± 22.8)	
**BMI ^d^ (kg/m^2^)**			
≥ 18.5	73	(42.5 ± 13.3)	0.733
< 18.5	8	(55.4 ± 42.4)	
**ASA-PS ^e^**			
1	52	(44.3 ± 19.9)	0.809
≥ 2	29	(42.9 ± 15.0)	
**GPS ^f^ score**			
0	69	(42.8 ± 13.6)	0.790
≥ 1	12	(49.7 ± 34.8)	
**PNI ^g^ score**			
≥ 40	77	(44.5 ± 18.4)	0.056
< 40	4	(31.0 ± 6.2)	
**pStage**			
II	47	(42.3 ± 14.1)	0.890
III	34	(45.8 ± 22.8)	
**Histological type**			
Differentiated	32	(42.3 ± 11.2)	0.761
Undifferentiated	49	(44.8 ± 21.6)	
**Surgical approach**			
Open	64	(45.4 ± 19.4)	0.196
Laparoscopy	17	37.8 ± 11.5)	
**Post-operative peak CRP ^h^ (mg/dl)**			
≥ 8.1	45	(47.8 ± 20.0)	**0.007**
< 8.1	36	(38.8 ± 14.3)	
**Complication (C-D^ i^ ≥ II)**			
Positive	12	(52.3 ± 16.4)	**0.026**
Negative	69	(42.3 ± 18.2)	

^a^ PIAC: postoperative duration until adjuvant chemotherapy, ^b^ Mann-Whitney U test analysis; ^c^ SD: standard deviation, ^d^ BMI: body mass index; ^e^ ASA-PS: Physical status proposed by the American Society of Anesthesiologists (ASA); ^f^ GPS: Glasgow prognostic score, ^g^ PNI: prognostic nutritional index; ^h^ CRP: C-reactive protein, ^i^ C-D: Clavien-Dindo classification.

**Table 4:** Univariate and multivariate analysis using Cox's proportional hazard model

Variable	n	Univariate ^a^	Multivariate ^b^		
		*p* value	HR^ c^	95% CI ^d^	*p-*value
Total	81				
**Sex**					
Male vs. Female	51 vs. 30	0.357			
**Age (years)**					
≥ 65 vs. < 65	43 vs. 38	0.776			
**BMI (kg/m^2^)**					
< 18.5 vs. ≥ 18.5	8 vs. 73	0.080			
**pStage^e^**					
pStage III vs. pStage II	34 vs. 47	**< 0.001**	3.08	1.41-7.22	**0.005**
**Histological type**					
Undifferentiated vs. Differentiated	49 vs. 32	0.856			
**Postoperative peak-CRP (mg/dl)**					
≥ 8.1 vs. < 8.1	45 vs. 36	0.658			
**Complication (C-D^f^ ≥ II)**					
Positive vs. Negative	12 vs. 69	0.480			
**PIAC (weeks)**					
≥ 7 vs. < 7	23 vs. 58	**0.004**	2.45	1.13-5.29	**0.024**
**Cumulative S-1 dose/BSA^g^ (mg/m^2^)**					
< 12,000 vs. ≥12,000	40 vs. 41	**0.002**	3.27	1.43-8.37	**0.004**

^a^ Analyzed by Log Rank (Mantel-Cox) test; ^b^ Analyzed by Cox's proportional hazard model; ^c^ HR: hazard ratio, ^d^ CI: confidence interval; ^e^ Japanese Classification of Gastric Carcinoma; ^f^ C-D: Clavien-Dindo classification, ^g^ BSA: body surface area.

## Abbreviations

GC: gastric cancer
AC: adjuvant chemotherapy
PIAC: postoperative duration until adjuvant chemotherapy
RFS: relapse-free survival
ACTS-GC: Adjuvant Chemotherapy Trial of TS-1 for Gastric Cancer
